# **Long-term outcomes after a trimalleolar fracture involving the posterior malleolar fragment: an 11-year follow-up**

**DOI:** 10.1007/s00402-025-05959-w

**Published:** 2025-06-18

**Authors:** Amal Chidda, Sérgio Soares, Moritz Tannast, Joseph Schwab, Angela Seidel

**Affiliations:** 1 Department of Orthopaedic Surgery and Traumatology, Fribourg Cantonal Hospital, University of Fribourg, Fribourg, Switzerland; 2 University Hospital of Bern, Bern, Switzerland

**Keywords:** Ankle fracture, Clinical outcomes, Post-traumatic ankle osteoarthritis, Posterior malleolar fracture, Volkmann’s fracture

## Abstract

**Introduction:**

We compared long term clinical and radiological outcomes of patients with trimalleolar ankle fractures including a posterior malleolar fragment (PMF) between those who underwent PMF fixation and those who did not. We also compared complication rates and identified independent risk factors associated with worse outcome.

**Material and Methods:**

We included 69 consecutive patients undergoing operation for a trimalleolar fracture between 2008 and 2013. Mean follow-up was 11.3 years. Patients completed the SF-12 and EFAS scores. Radiological osteoarthritis (OA) was assessed using the Kellgren-Lawrence classification. Postoperative complications were classified according to Sink. PMF size was measured on preoperative x-ray and CT and classified according to the Haraguchi classification.

**Results:**

The non-fixated group (*n* = 48) had a mean PCS score of 47.9, a mean MCS score of 54.1, a mean EFAS score of 17.1, and a mean EFAS-Sport score of 4.35. The fixated group (*n* = 21) had a mean PCS score of 49.2, a mean MCS score of 56.5, a mean EFAS score of 17.5, and a mean EFAS-Sport score of 6.05. There was no statistical difference between the two groups in the long term clinical PROMS. Patients in the fixated group developed more advanced OA (*p* = 0.013).

**Conclusion:**

Patients who underwent PMF fixation had a larger PMF size, more complications and more advanced OA, but with similar long term clinical outcomes than the non-fixated group.

**Level of Clinical Evidence:**

III, Retrospective comparative study.

## Introduction

Trimalleolar ankle fractures represent 10% of all ankle fractures [1] and are usually treated surgically [2]. In addition to medial and lateral malleoli fixation, a posterior malleolar fragment (PMF) can be fixed using percutaneous screws or through a separate posterolateral or posteromedial approach with plates or screws [3]. Trimalleolar fractures, regardless of whether or not the posterior malleolar fragment undergoes fixation, are known to have a worse outcome than bimalleolar and unimalleolar fracture [4].

Fixation of the PMF carries with it the possibility of technical challenges, increased surgical time, and wound complications, related to the posterolateral approach [5]. Although PMF fixation restores joint stability by stabilizing the posterior syndesmosis [6], it remains unclear whether if the complications associated with fixation offset the benefit of improved joint stability on long term outcomes. Several studies have assessed the postoperative complication rate at short and mid-term [7, 8], but not their long term impact. Moreover, PMF fixation does not seem to prevent OA development, which contributes to long term outcomes in these injuries [9].

This study does not aim to determine the indication for PMF fixation, but rather to compare the long-term outcomes associated with its fixation or non-fixation in clinical practice. We therefore aimed to investigate the long-term clinical and radiological outcomes of patients presenting with a trimalleolar fracture, comparing cases with and without fixation of the posterior malleolar fragment (PMF). Additionally, we examined the differences in postoperative complication rates between these two groups, identified independent risk factors associated with worse clinical and radiological outcomes, and analyzed the relationship between PMF size and long-term outcomes.

## Materials and methods

We obtained approval from the Ethics Committee (CER-VD, 2021 − 01120). For this retrospective comparative study, we identified 179 patients in two centers treated operatively for a trimalleolar ankle fracture with PMF between March 31, 2008 and December 5, 2013 who met the inclusion criteria. Inclusion and exclusion criteria are listed in Table 1.

**Table 1:** Inclusion and exclusion criteria

Inclusion criteria	• Patients aged above 18 years • Patients with operative treatment of trimalleolar ankle fractures with fixation of both the lateral and medial malleoli • Patient with sufficient cognitive and linguistic ability in order to participate in the study
Exclusion criteria	• Pathological fractures • Pilon fractures • Patient treated for a fracture at the same ankle before • Patient treated for the fracture outside of our hospital • Patient non ambulating before time of injury

**Fig. 1:**
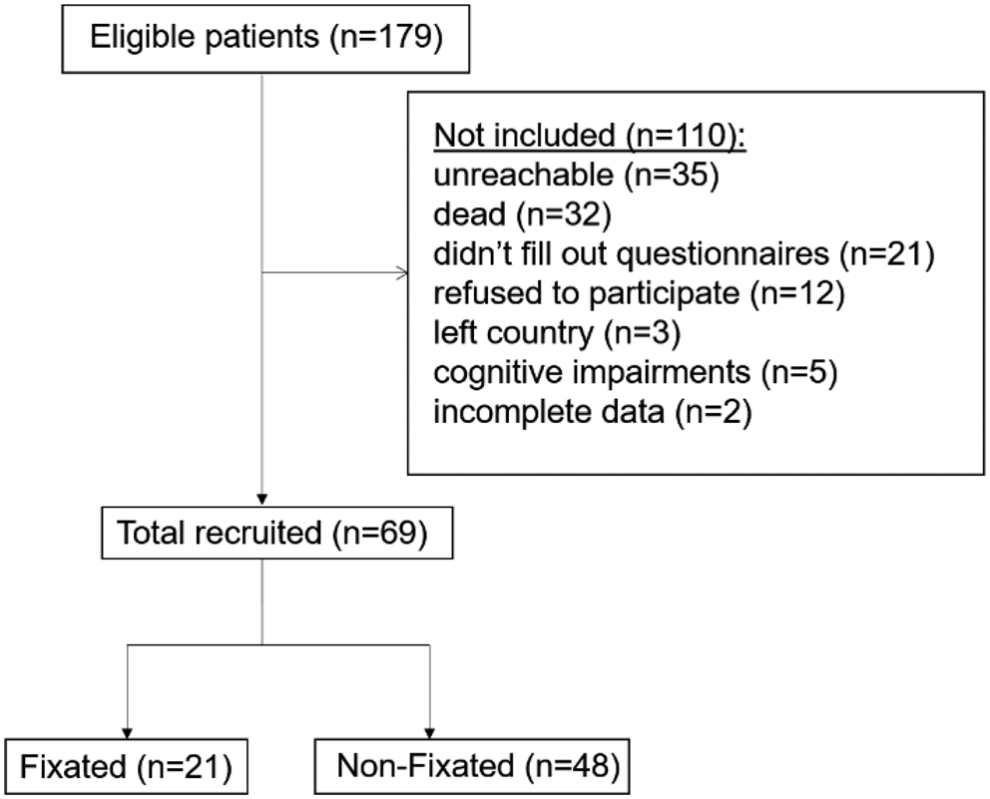
Flowchart illustrating the recruitment process

Patients were contacted by phone, and those who agreed to participate received PROMS by mail or email. Radiographic evaluations were performed for attendees, but non-attendees were also included. Of the 179 eligible patients, we recruited 69 patients. See Fig. 1.

Surgical treatment details were left up to each individual treating surgeon. Typically, the medial malleolus was exposed through a medial approach and fixed with screws, k-wires, tension band, or a combination of these. When the PMF was fixed, it was through a posterolateral approach to have access to both the fibula and the posterior tibia. Lateral malleolar fractures were fixed with screws, plates, k-wires, or a combination of these. For the PMF fixation, an antiglide plating construct was applied. When the PMF was not fixed, fibular fractures were exposed through lateral approach.

Postoperatively, patients were placed into a removable boot and allowed 5–15 kg weightbearing through their operative extremity, at the surgeon’s discretion, for approximately 6 weeks. Patients were given VTE prophylaxis for six weeks. Physical therapy was initiated at the surgeon’s discretion and in some cases began immediately after surgery, while in other cases it began after the 6-week follow-up.

Fractures were classified according to the Lauge-Hansen classification [10]. The follow-up appointment was conducted at approximately 10 years. Patient-reported outcome measures, including the SF-12 and EFAS scores, were completed either during this appointment or returned by mail. The SF12 is a validated health-related quality of life score used in different health domains that comprises a Physical Component Summary (PCS) and a Mental Component Summary (MCS) score [11, 12]. The EFAS score is a validated score for foot and ankle pathologies [13]. The methodology, including data collection, radiographic assessments, and outcome measures, follows previously described methods [14]. Postoperative complications were categorized according to the Sink classification, which categorizes complications based on severity and impact on patient management, from Grade I (minor) to Grade V (death) [15].

**Fig. 2:**
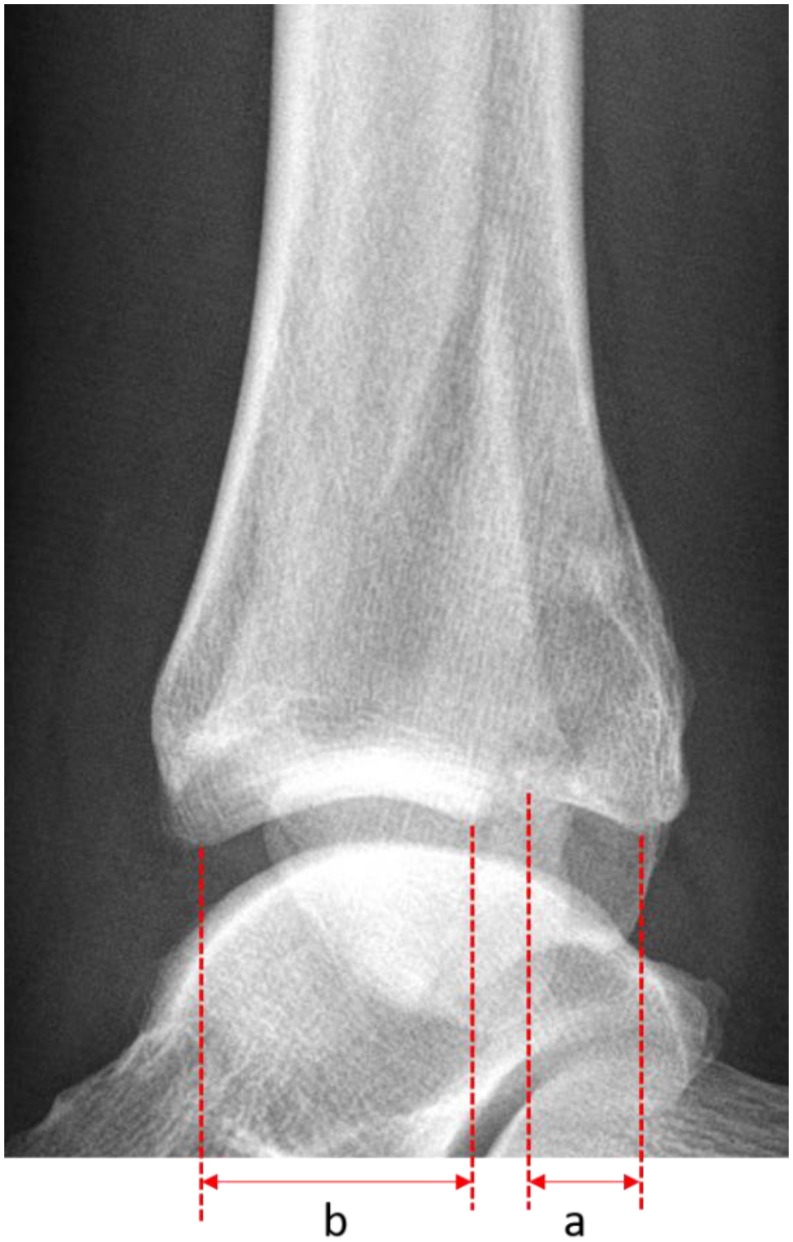
The size of the PMF is measured in the lateral X-ray and divided by the total articular surface then expressed in percent

We measured the PMF size in the preoperative lateral radiograph of the ankle in 53 patients, calculating it as a percentage of the distal tibial articular surface using the PACS system (Fig. 2) [9].

**Fig. 3:**
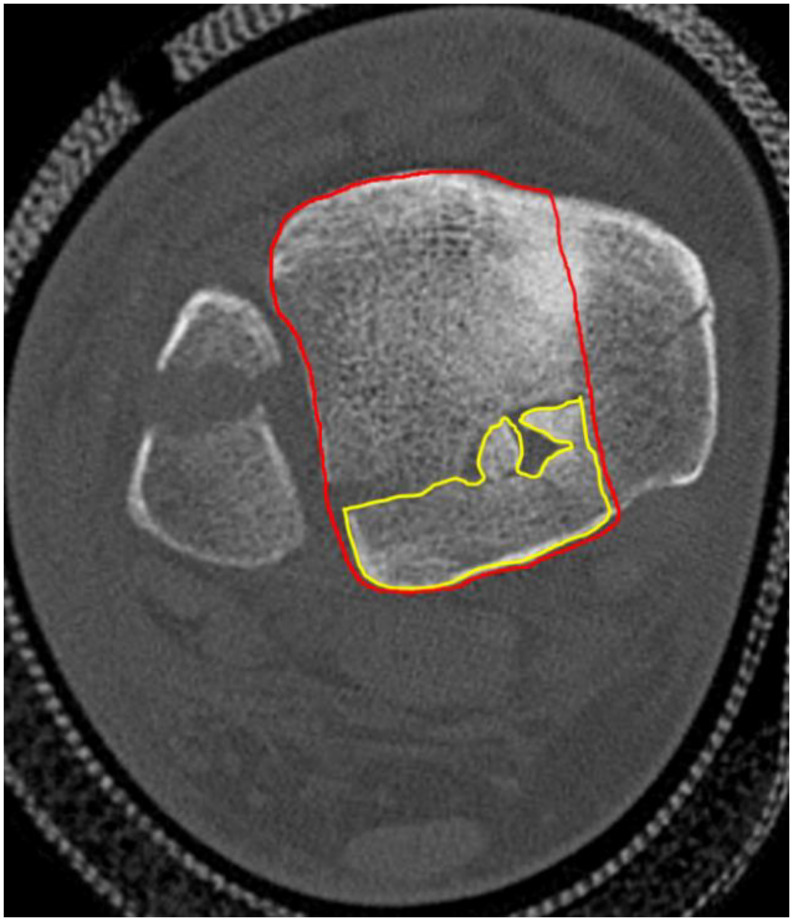
In the method 1, the PMF area is divided by the total articular surface of the tibia minus the medial malleolus on the axial view of the CT then expressed in %

**Fig. 4:**
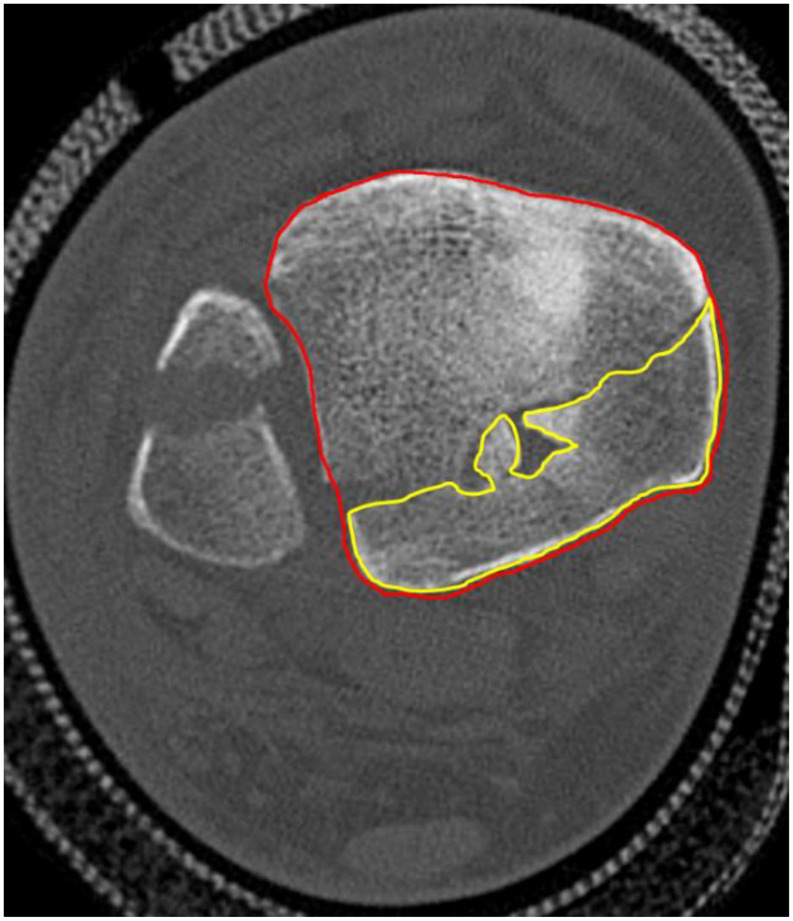
In the method 2 the PMF area is divided by the total articular surface of the tibia on the axial view of the CT then expressed in %

For the patients (*n* = 18) who had a preoperative CT of the ankle we classified the PMF according to the Haraguchi classification [16]. The PMF surface was measured in two ways on axial views: as a percentage of the distal tibia surface excluding the medial malleolus (Fig. 3) [17] and including it (Fig. 4) [7]. Measurements were taken at the slice with the maximum PMF diameter

We evaluated osteoarthritis in 44 patients using ankle radiographs and classified it according to the Kellgren and Lawrence classification [18]. We defined moderate OA as Kellgren and Lawrence stages I and II; Advanced OA was defined as Kellgren and Lawrence stages III and IV [19].

### Statistical analysis

Statistical analysis was performed with R and R Studio. (R Core Team [2023]. R: A Language and Environment for Statistical Computing. R Foundation for Statistical Computing, Vienna, Austria. https://www.R-project.org/). Non-parametric tests were used (Wilcoxon rank sum test, Fisher’s Exact test) and regression analysis. Statistical significance was defined at *p* ≤ 0.05.

## Results

### Demographics

69 patients of 179 patients with tri-malleolar fractures were included (Table 2). Twenty-one (30.4%) patients had undergone plate fixation of the posterior malleolus and 48 (69.6%) had no fixation of the posterior malleolus. 49 patients were female (71%) and 20 (29%) were male. Mean age was 51.4years and mean BMI was 26.7. Nine patients (13%) had diabetes and 20 patients (29.4%) were smokers. The demographics were similar between the two groups except for the smoking history (*p* = 0.028). Table 2. Demographics.

**Table 2:** Demographics

		Non-fixated, *n* = 48	Fixated, *n* = 21	Total, *n* = 69	*p*-value
**Gender**					0.075
	Male	17 (35%)	3 (14%)	20 (29%)	-
	Female	31 (65%)	18 (86%)	49 (71%)	-
**Age (years)**		52.0 ± 14.8	49.8 ± 15.05	51.4 ± 14.5	0.4
**BMI (kg /m2)**		26.4 ± 4.61	27.15 ± 5.29	26.7 ± 4.58	0.6
**ASA**					0.2
	I	14 (31%)	4 (19%)	18 (26.1%)	-
	II	27 (60%)	17 (81%)	44 (63.8%)	-
	III	4 (8.9%)	0	4 (5.8%)	-
**Smoking history**	Yes	10 (21%)	10 (48%)	20 (29%)	**0.028**
**Diabetes**	Yes	8 (17%)	1 (4.8%)	9 (13.0%)	0.3
**Mean follow-up time (years)**		11.4 ± 1.38	11.04 ± 1.82	11.3 ± 1.38	0.8

Values are given either as total number (percentage) or mean ± standard deviation. The percentages are the ratio between the number and the total number of the column.

## Long term clinical outcomes

**Fig. 5:**
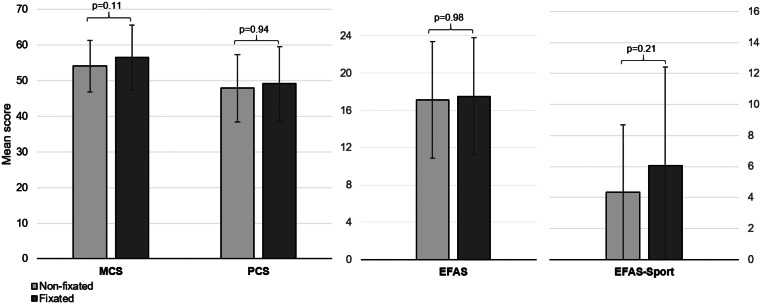
Mean score of MCS, PCS, EFAS and EFAS-Sport with the SD

The non-fixated group had a mean of PCS of 47.9 ± 9.4 and the fixated group had a mean of PCS of 49.2 ± 10.4 (*p* = 0.94). The non-fixated group had a mean of MCS of 54.1 ± 7.21 and the fixated group had a mean of MCS of 56.5 ± 9.09 (*p* = 0.11). The non-fixated group had a mean of EFAS of 17.1 ± 6.27 and the fixated group had a mean of EFAS of 17.5 ± 6.31 (*p* = 0.98). The non-fixated group had a mean of EFAS-Sport of 4.35 ± 5.76 and the fixated group had a mean of EFAS-Sport of 6.05 ± 5.82 (*p* = 0.21). See Fig. 5.

## Long term radiological outcomes

**Fig. 6:**
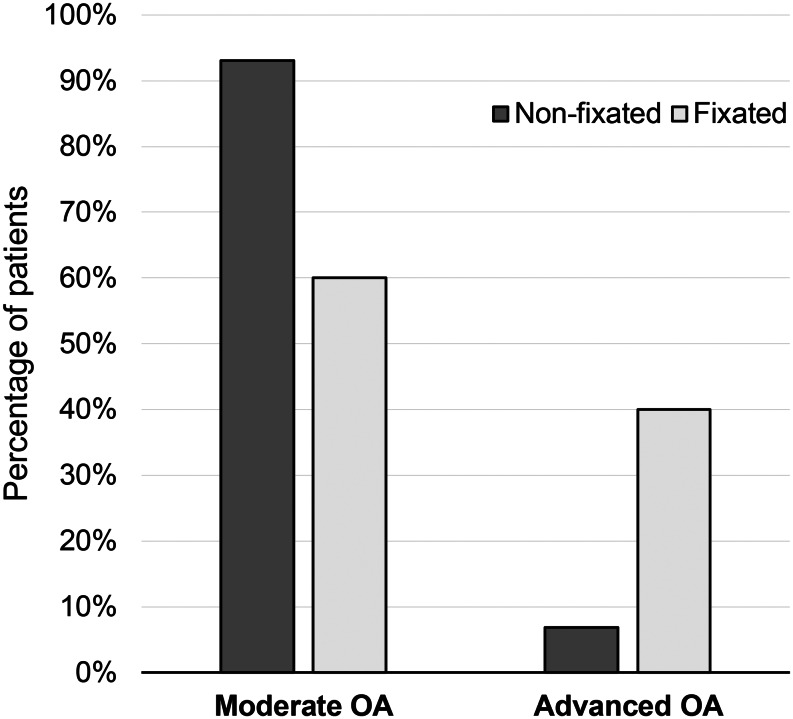
Moderate OA corresponds to grade I and II of the Kellgren and Lawrence classifcation and Severe OA to grade III and IV

Forty-four patients presented for long-term radiographic evaluation. There were 15 patients in the fixated group and 29 patients in the non-fixated group. Nine (60%) patients in the fixated group versus 27 (93%) patients in the non-fixated group were graded as Moderate OA, and 6 (40%) patients in the fixated group versus 2 (6.9%) patients in the non-fixated group were graded as Advanced OA (*p* = 0.013). See Fig. 6.

### Complication rate

Patients in the non-fixated group had no postoperative complications. Six patients in the fixated group (28.6%) had a postoperative complication (*p* < 0.001). Complications consisted of two wound lesions, one infection, one CRPS, one nerve lesion and one non-union. See Table 3.

**Table 3:** Postoperative complications

		Non-fixated, *n* = 48	Fixated, *n* = 21	Total, *n* = 69	*p*-value
**Postoperative Complications**	Yes	0	6 (29%)	6 (8.7%)	**< 0.001**
**Sink Classification**					> 0.9
	II	0	2	2 (2.9%)	-
	III	0	4	4 (5.8%)	-

The percentages are the ratio between the number and the total number of the column.

## Independent predictors

In the linear regression analysis, we found that diabetes was a predictor of negative outcome for the PCS (HR=-7.7.0 [95% CI -15 to − 0.25]; *p* = 0.043), EFAS (HR=-5.5 [95%CI -10 to -0.62]l *p* = 0.028) and EFAS Sport (HR=-5.8 [95%CI -10 to -1.3]; *p* = 0.013). Dislocation was also a predictor of negative outcome for the EFAS score (HR=-4.1 [95%CI -7.3 to -0.86]; *p* = 0.014). No predictors of osteoarthritis were identified.

Values are given either as total number (percentage) or mean ± standard deviation. The percentages are the ratio between the number and the total number of the column. Abbreviations: SER: Supination External Rotation, PA: Pronation Abduction, PER: Pronation External Rotation.

PMF size was significantly larger in the fixated group across all measurement methods. No significant differences were found between groups regarding fracture classification (Weber, Lauge-Hansen, Haraguchi), nor presence of dislocation.

PMF size was negatively correlated with the mental part of the SF-12 (*p* = 0.03), but not to any other measurements of long-term clinical outcome. (Table 4)

**Table 4:** Fracture description

		Non-fixated, *n* = 48	Fixated, *n* = 21	Total, *n* = 69	*p*-value
**Dislocated fracture**					0.1
	Yes	23 (47.9%)	15 (71%)	38 (55%)	-
	No	23 (47.9%)	6 (29%)	29 (42%)	-
**Weber Classification**					0.1
	B	44 (91.7%)	16 (76%)	60 (87%)	-
	C	3 (6.3%)	5 (24%)	8 (11.6%)	-
**Lauge-Hansen classification**					0.2
	SER IV	40 (83.3%)	18 (90%)	58 (84%)	-
	PA III	3 (6.3%)	0	3 (4.35%)	-
	PER IV	1 (2.08%)	2 (10%)	3 (4.35%)	-
**Haraguchi classification**					0.085
	I	3	1	4	-
	II	2	8	10	-
	III	3	1	4	-
**Measurement of PMF size (in %)**					
	On lateral x-ray	20.6 ± 8	30.1 ± 8	23.2 ± 8	**< 0.001**
	On CT (method 1)	7.68 ± 7	18.5 ± 7	13.7 ± 7	**< 0.001**
	On CT (method 2)	8.16 ± 10	21.6 ± 9	15.6 ± 10	**< 0.001**

Values are given either as total number (percentage) or mean ± standard deviation. The percentages are the ratio between the number and the total number of the column. Abbreviations: SER: Supination External Rotation, PA: Pronation Abduction, PER: Pronation External Rotation.

## Follow-up surgeries

**Fig. 7:**
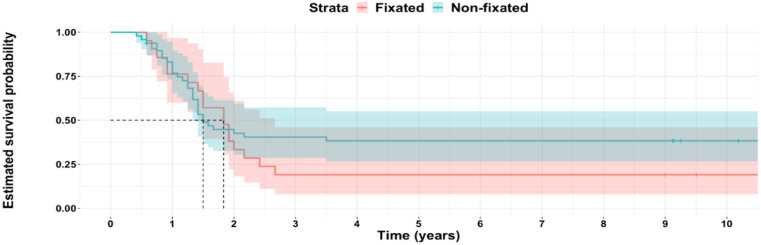
Survival probability from any subsequent surgery

Forty-seven patients underwent follow-up surgeries, see Table 5. (Fig. 7)

**Table 5:** Follow-up surgeries

		Non-fixated, *n* = 48	Fixated, *n* = 21	Total, *n* = 69	*p*-value
**Follow-up surgery**		30 (62.5%)	17 (81%)	47 (68.1%)	0.13
	Hardware removal	29 (60.4%)	16 (76%)	45	0.2
	Arthrodesis	1 (2.08%)	1 (4.8%)	2	0.5
	Total ankle replacement	0	1 (4.8%)	1	0.3

The percentages represent the ratio between the patients who underwent follow-up surgery divided by the total number of patients in each group. Figure 8 shows the survivorship curve free from any subsequent surgery: at 10 years, 19% of the fixated group remain without surgery and 38% of the non-fixated group (*p* = 0.3).

## Discussion

In our study, long-term clinical outcomes for patients without PMF fixation were similar to those with fixation, although there were notable differences between the two groups. In particular, the surgical approach differed and the PMF size was smaller in the non-fixated group. This suggests that fixation decision at the time were primarly based on fragment size, whereas current evidence emphasizes the greater relevance of PMF morphology assessed on CT. De Vries [20] also evaluated the long-term outcomes of 45 patients with trimalleolar fractures and found similar outcomes between the fixated and non-fixated group. Likewise, the PMF size was significantly smaller in the non-fixated group than in the fixated group (16% versus 30%).

In a retrospective study with a mean follow-up of 33.5months, patients who underwent PMF fixation had a better clinical outcome [21]. While their demographics were similar, the fixed group had a higher proportion of Mason 3 type PMF, considered most severe grade of PMF fracture [22].

In a recent systematic review [23], PMF fixation showed no statistical difference in AOFAS score compared to the untreated PMF (*p* = 0.21), despite higher scores in the fixated group. Moreover, a recent prospective randomized study [24] showed no statistical difference in functional and radiological outcome between the fixed versus non fixed middle-sized (10–25%) PMF in the short to mid-term follow-up. These results suggest that fixation of the PMF does not necessarily leads to better outcomes and that it should be carefully assessed. Since our study has a retrospective design and we could not control for PMF fixation decision, our study does not resolve the question about what the size cutoff for PMF fixation may be.

Both groups had a mental quality of life (mean MCS = 54.9) exceeding the Swiss norm of 46.3 [25]. However, their physical quality of life (mean PCS = 48.3) was slightly lower the Swiss norm of 49.8 [25]. Overall, their quality of life outcomes aligns with the general population, indicating favorable outcomes after a trimalleolar fracture.

Patients with fixation of the PMF and thus with a posterolateral approach for fixation had a complication rate of 28%, which is significantly higher that the unfixed group. Two thirds of them needed a secondary surgery. This increased morbidity is similar to Aravindan’s results who found that ORIF of PMF was associated with a higher wound complication rate (26%) [7]. Also, they found that the operative and tourniquet time were longer, and the blood loss was more significant compared to the group who did not have the PMF fixed [7]. This is showing that there is a correlation between the complication rate and the PMF fixation. But this correlation can be due to secondary confounders influencing both as the posterolateral incision, a more severe fracture needing a longer time. It is remarkable that even with the higher complication rate, the outcome at 10 years was similar in the two groups. A possible reason for the higher morbidity may be the posterolateral approach, which has a wound complication rate from 9.6% to up 44% [26, 27].

OA is a known complication of ankle fractures, and we observed eight patients (22%) with advanced OA after a mean follow-up of 11.3 years. Lübbeke et al. [19] found that 36% of patients with an operated malleolar fracture developed advanced OA after a mean follow-up of 17.9 years. In their systematic review, Swierstra et al. [28] found an incidence of 25% of post-traumatic OA and 34% in ankle fractures involving the PMF with a mean follow up of 6.75 years. In our study, patients with PMF fixation developed more advanced OA. However, we must acknowledge that this observation is based on a small number of patients with available radiographic follow-up and thus this result should be interpreted with caution.

It would be interesting if this association stays the same 20 years after injury. Ankle posttraumatic osteoarthritis development is multifactorial, and its exact mechanism is still unknown [29]. It might be due to a higher initial impact at trauma. More advanced age and persistent postoperative step-off of the PMF [9] are potential risk factors.

Our study aimed to detect predictors of worse outcomes after trimalleolar fracture. Diabetes was identified as an independent predictor of negative outcome in the PCS (physical component of SF12 score), EFAS and EFAS-sport scores. Diabetes is known to increase postoperative complications rate [8, 30] and predict poor short- and mid-term outcomes after ankle fractures [31, 32]. Our results show that diabetes also negatively affects long-term clinical outcomes.

Although PMF size is often considered during surgical planning, its clinical relevance has been increasingly questioned. In our study, PMF size was not markedly associated with the long-term clinical outcomes. In his systematic review, Odak [33] concluded that outcomes of ankle fractures were not related to PMF size but rather to factors like fracture dislocation at injury, the congruency of the articular surface, and residual tibiotalar subluxation.

A recent study [34] found no association between PMF size and worse clinical outcome. The PMF size may not have an influence in the long term clinical and radiological outcomes after a trimalleolar fracture if the ankle joint is congruent. New studies suggest that the morphology of the PMF has more prognostic importance than its size [35] and as a result several CT classifications have emerged. The Haraguchi classification is the first and most widely used CT-based classification [35]. According to Patel et al., Haraguchi type I fractures have a better outcome than type II and III fractures [36]. Although our study indicated no statistical difference in the Haraguchi classification distribution, 80% of the patients in the fixated group presented a Haraguchi type II fracture. A study with a higher number of preoperative CTs is needed to correlate PMF morphology with the long-term outcomes as it was not performed routinely in our department at that time. PMF size was overestimated in the lateral radiograph, which reinforces the idea that radiographs do not provide an exact accurate estimation of the PMF size [35] and should not be used alone as an indication for PMF fixation.

One of the limitations of this study is the lack of availability of preoperative x-ray or a preoperative CT-scan in some patients. While these patients had preoperative imaging, imaging is only reliably available in our informatics system since 2012. In addition, preoperative CT-scan has not been performed systematically at our institution. For those patients, the PMF size could not be evaluated in the preoperative imagery which may introduce bias in our results. Moreover, some patients did not have a long-term radiograph to estimate the osteoarthritis because of non-response to our requests for radiographic follow-up, which may have also introduced bias; we decided to include them because the main aim was to evaluate the long term clinical outcome. Another limitation is the retrospective design of the study which could not allow us to control for all factors including the decision to fixate the PMF, the type of fixation used, and variations in the postoperative protocol. Finally, the number of eligible patients who were lost to follow up or refused to participate in the study is a limitation that could have resulted in bias. The strength of the study is the long-term follow-up of the patients with an average above 10 years.

## Conclusion

In conclusion, patients in the fixation group had larger posterior fragments, more complications, and more advanced osteoarthritis, but these factors did not translate into worse long-term patient-reported outcomes. As the decision to fix the PM fragment was not standardized and likely reflected fracture severity and surgeon preference, these findings should not be interpreted as evidence that fixation is unnecessary.

## Data Availability

No datasets were generated or analysed during the current study.
